# Prognosis of Patients with Hepatocellular Carcinoma. Validation and Ranking of Established Staging-Systems in a Large Western HCC-Cohort

**DOI:** 10.1371/journal.pone.0045066

**Published:** 2012-10-05

**Authors:** Mark op den Winkel, Dorothea Nagel, Julia Sappl, Philip op den Winkel, Rolf Lamerz, Christoph J. Zech, Gundula Straub, Thomas Nickel, Markus Rentsch, Petra Stieber, Burkhard Göke, Frank T. Kolligs

**Affiliations:** 1 Department of Medicine II, Campus Grosshadern, University of Munich, Germany; 2 Institute of Clinical Chemistry, Campus Grosshadern, University of Munich, Germany; 3 Department of Clinical Radiology, Campus Grosshadern, University of Munich, Germany; 4 Department of Medicine I, Campus Grosshadern, University of Munich, Germany; 5 Department of Surgery, Campus Grosshadern, University of Munich, Germany; University Hospital Carl Gustav Carus, Germany

## Abstract

**Background:**

HCC is diagnosed in approximately half a million people per year, worldwide. Staging is a more complex issue than in most other cancer entities and, mainly due to unique geographic characteristics of the disease, no universally accepted staging system exists to date. Focusing on survival rates we analyzed demographic, etiological, clinical, laboratory and tumor characteristics of HCC-patients in our institution and applied the common staging systems. Furthermore we aimed at identifying the most suitable of the current staging systems for predicting survival.

**Methodology/Principal Findings:**

Overall, 405 patients with HCC were identified from an electronic medical record database. The following seven staging systems were applied and ranked according to their ability to predict survival by using the Akaike information criterion (AIC) and the concordance-index (c-index): BCLC, CLIP, GETCH, JIS, Okuda, TNM and Child-Pugh. Separately, every single variable of each staging system was tested for prognostic meaning in uni- and multivariate analysis. Alcoholic cirrhosis (44.4%) was the leading etiological factor followed by viral hepatitis C (18.8%). Median survival was 18.1 months (95%-CI: 15.2–22.2). Ascites, bilirubin, alkaline phosphatase, AFP, number of tumor nodes and the BCLC tumor extension remained independent prognostic factors in multivariate analysis. Overall, all of the tested staging systems showed a reasonable discriminatory ability. CLIP (closely followed by JIS) was the top-ranked score in terms of prognostic capability with the best values of the AIC and c-index (AIC 2286, c-index 0.71), surpassing other established staging systems like BCLC (AIC 2343, c-index 0.66). The unidimensional scores TNM (AIC 2342, c-index 0.64) and Child-Pugh (AIC 2369, c-index 0.63) performed in an inferior fashion.

**Conclusions/Significance:**

Compared with six other staging systems, the CLIP-score was identified as the most suitable staging system for predicting prognosis in a large German cohort of predominantly non-surgical HCC-patients.

## Introduction

Hepatocellular carcinoma (HCC) is the fifth most common cancer worldwide [1], with the highest incidence in Asian and developing countries [2]. Still, especially when considering its rising incidence in the western world due to viral hepatitis and alcohol-induced cirrhosis [3], HCC is an important health issue in these geographic regions, as well. It is an aggressive tumor making it the third most common cause of cancer related death worldwide [4]. In approximately 80–90% of all HCC-cases, liver cirrhosis forms the underlying precancerosis that favors tumor development. Tumor-staging, prognosis-estimation and choosing of treatment options for HCC patients is a more complex issue than in most other cancer-entities. This is due to the fact that the extent of liver dysfunction has a major impact on survival, sometimes more than the tumor itself. This is why the Child-Pugh score, although not being an HCC staging system in its actual sense, has been used to stratify HCC patients as well. Nevertheless, traditional uni-dimensional classifications like the TNM-system [5] or the Child-Pugh-score [6], exclusively taking into account tumor stage or liver dysfunction, respectively, do not account for the complexity of HCC in cirrhosis. As a consequence, multidimensional staging systems which include both the extension of tumor and liver function parameters (sometimes plus general health variables) have been developed: Okuda [7], Barcelona Clinic Liver Cancer (BCLC) [8], Cancer of the Liver Italian Program (CLIP) [9], Groupe d'Etude et de Traitement du Carcinome Hépatocellulaire (GETCH) [10] and Japan Integrated Staging (JIS) [11] [**For details, see supporting information tables S1, S2, S3, S4, S5, S6, S7, S8**]. It has been claimed, that linking staging with treatment decisions is mandatory [12]. The only staging system currently providing this linkage is BCLC. Therefore, BCLC has been endorsed as the recommended staging system by American and European medical societies [13], [14]. Despite this, BCLC has been criticized for being too algorithmic. In various studies it has performed in an inferior fashion especially when applied to non-surgical patients [15] and in some studies even when applied to surgical patients [16].

After all, it remains unclear which of the established staging systems should be preferred for a patient diagnosed with HCC. A precise answer to this question would facilitate not only clinical management of the individual patient but risk stratification in clinical studies, as well. This is a critical issue since a rising number of clinical studies can be noted due to the advent of effective systemic treatment options [17]. It has been suggested, that the consistent use of validated staging systems could help improving the overall grim prognosis of HCC [18]. Nevertheless, efforts to construct a universally applicable staging system are doomed to fail because this approach would neglect the unique geographic characteristics of HCC, including epidemiological and etiological parameters. Therefore, a more region-oriented approach seems necessary, with validation of the established staging systems within the context of the specific geographic disease background.

### Objectives

The aim of this study was to compare the ability of seven established staging systems to predict survival for patients in a large western HCC population. The validation of the staging systems was preceded by a precise retrospective characterization of the study population in order to ensure proper interpretation of the validation data. Additionally, this analysis was designed to identify the most relevant single prognostic variables incorporated in the staging systems.

## Patients and Methods

### Patients

In this retrospective study, we identified HCC- patients treated at the Department of Medicine II of Munich's University Hospital between January 1998 and March 2009. The research study was approved by the ethics committee of the University of Munich and the need for written informed consent was waived, because the data were analyzed retrospectively and anonymously. Histological or radiological (AASLD radiologic criteria [19]) confirmation of diagnosis was mandatory for inclusion. Baseline was defined as time of primary diagnosis of HCC, and certain baseline examinations including laboratory and imaging studies were required for inclusion in the study. Patients were excluded when showing too fragmentary documentation of the data (>4 parameters missing) or whenever the survival status was unknown. In total, 550 consecutive patients with HCC were identified, of these 145 had to be excluded because of lacking data, leaving a study population of 405 patients.

### Data Collection

Patients were identified from a data base collection in our institution, by using the International Classification of Diseases (ICD) code 150.0 for primary liver cancer. Clinical, tumor related and laboratory data needed to stage patients in all seven staging systems were retrieved from our electronic medical records. Additionally, a wide range of other parameters was compiled in order to further characterize our HCC-collective. The following data were collected: Age, sex, date of initial diagnosis, date of initial therapy, survival status, date of death, end of observation, liver cirrhosis, etiology, mode of therapy, Eastern Cooperative Oncology Group status (ECOG), Karnofsky-index, histology, ascites, hepatic encephalopathy (HE), portal vein thrombosis, portal hypertension, tumor extension, tumor burden (>/<50% of liver), number of tumor nodes, macroscopic vascular invasion, distant metastasis, lymph node involvement, BCLC tumor features ([1]: singular <2 cm, [2]: 3 nodules ≤3 cm or 1 nodule 2- ≤5 cm, [3]: multilocular, [4]: Portal invasion, N1, M1). Furthermore, the following laboratory parameters were retrieved in order to be able to calculate all tested staging systems: AFP, bilirubin, alkaline phosphatase, Quick and albumin.

In those cases without histology, the diagnosis of liver cirrhosis was made dependent on typical clinical signs of portal hypertension or on unequivocal radiological signs. Portal hypertension was diagnosed, if an elevated hepatic vein pressure above 10 mm/Hg, esophageal varices, splenomegaly or a platelet count below 100.000/µl were noted. Classification of ascites was performed according to the Child-Pugh score. Ascites detected by imaging but not visible on physical examination was termed mild, while the ascites was classified as “massive”, if clinically visible. Whenever exact classification of HE was missing in medical records, clinical signs of HE like tiredness, confusion and coma were used to retrospectively classify the respective HE grades I–IV [20].

Whenever medical records did not include exact documentation of Karnofsky performance (KPS) *and* Eastern Cooperative Oncology Group performance status (ECOG), these classifications were retrospectively estimated on the basis of the available data on the general health status of the patient. For patients with exact documentation of either KPS *or* ECOG, the missing score was deducted on the basis of the following estimation [21]: ECOG 0 = KPS 100%, ECOG 1 = KPS 80%–90%, ECOG 2 = KPS 60%–70%, ECOG 3 = KPS 40%–50% and ECOG 4 = KPS 10%–30%.

All treatment decisions were based on an interdisciplinary tumor composed of hepatologists, (interventional-) radiologists, oncologists and surgeons. Although the advent of staging systems including treatment recommendations according to specific stages like BCLC has had an impact on these boards, treatment allocation to date remains an individual approach.

All baseline tumor parameters necessary to characterize the HCC-cohort and to calculate the staging systems were obtained by reviewing radiology and pathology reports, respectively. When in doubt concerning certain tumor measurements a radiologist (C.Z.) with 8 years experience in abdominal CT and MRI reevaluated the baseline images. Regional lymph node involvement was assumed when suspect lymph nodes (>1 cm in diameter) were detected on MRI and CT, respectively. Information on survival was retrieved from the clinical records, whenever possible. In all other cases the primary care physician was contacted via telephone or fax.

### Staging Systems

Out of 405, 365 patients showed sufficient data to perform stratification according to Child-Pugh-score, 395 patients according to TNM, 373 patients according to Okuda, 352 patients according to CLIP, 341 patients according to BCLC, 358 patients according to JIS, and 304 patients according to GETCH. 290 Patients could be classified by all staging systems. In order to keep the numbers of patients with incomplete data as small as possible this cohort was enlarged to 354 patients by substituting missing values for laboratory parameters by the median (Bilirubin 1, Quick 2, AFP 11, Albumin 16, and AP 42 values). Ranking of scores was done for both cohorts of 290 and 354 patients, respectively. There were no substantial differences found, thus only values for the 354 patients are reported.

### Statistical Analysis

For statistical analysis SAS-Software [SAS V9.2, SAS Institute Inc., Cary, NC] was used. p<0.05 indicated statistical significance, with a p<0.0001 the parameter was considered to be of high statistical significance.

### Univariate analysis

For univariate analysis overall survival was estimated by using the Kaplan-Meier method from the date of primary diagnosis of HCC to the date of death or last follow-up. Survival curves were compared using the log-rank test. Additionally to the p-value medians of survival time and 95% confidence intervals for the different strata are given. Both, single parameters and the whole scores were analysed concerning their prognostic significance. For Kaplan-Meier-analysis of continuous variables, one or more cut-off values are necessary; therefore, laboratory values were divided into quartiles.

### Multivariate analysis

While the univariate analysis was performed for all the patients showing the individual parameter, multivariate analysis relates only to the cohort of n = 354 patients who could be classified in all staging systems as described above. This number reflects those patients who could be classified in all staging systems. In order to keep the numbers of patients with incomplete data as small as possible, for calculating the scores and for multivariate analysis missing values for laboratory parameters were substituted by the median. In those parameters showing significance in univariate analysis using Cox proportional hazards regression model was conducted in order to examine their independent prognostic relevance. To avoid arbitrary cut-off values in this model laboratory values were taken as base two logarithms and used as continuous variables.

### Ranking

Ranking of staging systems was achieved by the Akaike information criterion (AIC) [22] derived from the Cox model and concordance- index (c-index) [23]. AIC is a measure of relative goodness-of-fit and thus provides a means for comparing models, a lower AIC value indicating a better model fit. Calculating the c-index requires no model assumptions, it represents the proportion of concordance in all possible pairs of patients meaning that the patient with the better prognostic score has the longer survival time. A score with a c-index of 0.5 is not better than chance, a c-index of 1 indicates perfect prediction. C-indices together with 95% confidence intervals were calculated using the SAS macro [24]. In cases with disconcordant values of AIC and c-index, the AIC-value was favoured.

## Results

### Etiological factors

The etiological factors for HCC are reported in **table 1**. The sole leading etiological factor was alcohol abuse in 180 (44.4%) patients. Chronic viral hepatitis C or B were found in 100 patients (24.7%), with HCV being more frequent than HBV (76 (18.8%) and 24 (5.9%), respectively). In 14.8% of all cases no etiological factor could be identified, therefore these cases were classified as “cryptogenic”. 23 (5.7%) patients had other established, yet less common HCC etiologies. In 52 patients (10.3%) a combination of 2 etiological factors had contributed to HCC-development. The most frequent combination (21 patients (5.2%)) comprised the two most common single factors alcohol and HCV. When taking into account the cases of combined etiology, alcohol was noted in 212 (52.3%) and viral hepatitis in 138 (34.1%) cases.

**Table 1:** Etiology.

Etiological Factor	n	%
**Alcohol**	180	44.4
**HCV**	76	18.8
**Cryptogenic**	60	14.8
**HBV**	24	5.9
**Others:**	23	5.7
*Hemochromatosis*	10	
*Autoimmune hepatitis*	3	
*PBC*	3	
*Toxic*	2	
*Caroli-syndrome*	1	
*NASH*	1	
*PSC*	1	
*Tyrosinemia*	1	
*Alpha-1-Antitrypsin-Deficiency*	1	
**HCV and Alcohol**	21	5.2
**HBV and Alcohol**	7	1.7
**HCV and HBV**	4	1.0
**Alcohol and others:**	4	1.0
*Hemochromatosis*	3	
*Morbus Wilson*	1	
**HCV and others:**	3	0.7
*Hemochromatosis*	1	
*PBC*	1	
*Toxic*	1	
**HBV and others:**	3	0.7
*HDV*	3	

### Demographic and clinical data

Diagnosis of HCC was based on histology in 52.1% of patients. The most relevant clinical and demographic data of the patient population are depicted in **table 2**. With 335 patients the majority of patients were male (82.3%). The median age of all patients was 63.4 years (range 27.8–84.8). With 64.1 years (range 27.8–84.8) (female) vs. 63.3 years (28.0–84.6) (male), the age at time of primary diagnosis showed no relevant difference between both sexes. Liver cirrhosis as an underlying condition for HCC development was present in 338 patients (83.7%). As a consequence of liver cirrhosis 247 (63.7%) patients showed signs of portal hypertension at time of HCC diagnosis. Ascites was not present in the majority of patients (66.5%), the same was true for hepatic encephalopathy (HE) (77.4% without HE). Liver function was compensated (no cirrhosis or Child A cirrhosis) in more than half of the patients (53.7%), only 43 patients (13.4%) had Child-Pugh C end stage liver disease. Consistently, most of the patients were in a good or fairly good general condition at time of HCC-diagnosis, with 334 (92.6%) presenting with an ECOG of 0–1.

**Table 2:** Baseline demographic and clinical parameters.

	n	%	Median survival [months]	95%-CI	p-value
**Age**					0.163
<64 Years	199	49.1	15.5	12.2–18.8	
>64 Years	206	50.9	23.1	18.1–29.7	
**Sex**					0.3872
Female	70	17.3	19.6	14.4–32.7	
Male	335	82.7	17.2	14.4–21.7	
**ECOG**					<0.0001*
0	219	60.7	22.9	16.9–28.8	0 vs. 1: 0.002*
1	115	31.9	13.7	9.8–20.3	1 vs. 2: 0.061
2	21	5.8	3.9	2.1–23.1	2 vs. 3: 0.108
3	6	1.7	1.6	0.5–8.4	
**Liver cirrhosis**					0.0417*
No	66	16.3	28.4	18.9–38.2	
Yes	338	83.7	16.1	14.1–21.3	
**Ascites**					<0.0001*
No	266	66.5	25.6	21.1–29.7	No vs. mo:<0.0001*
Moderate	89	22.3	11,.1	7.3–15.2	Mo. vs. ma: <0.0001*
Massive	45	11.3	3.3	2.3–4.5	
**Hepatic Encephalopathy**					0.1605
No	291	77.4	20.1	15.6–24.1	
Yes	85	22.6	11.7	7.6–21.4	
**Portal Hypertension**					0.0310*
No	141	36.3	25.6	15.5–30.8	
Yes	247	63.7	16.1	14.1–20.6	
**Portal vein thrombosis**					<0.0001*
No	327	81.6	21.4	17.2–25.6	No vs. part: <0.0001*
Partial	54	13.5	6.0	3.9–15.2	Part. vs. comp: 0.182
Complete	20	5.0	4.4	1.9–9.2	

(* = statistically significant). Mo = moderate, ma = massive, part = partial, comp = complete.

### Laboratory parameters

The results of the evaluation of baseline laboratory parameters that are part of some of the tested staging systems are summarized in **table 3**. While AFP (40.5 ng/ml), aP (142 U/l) and bilirubin (1.3 mg/dl) showed elevated median values, Quick (75%) and albumin (3.8 g/dl) were within normal range. All 5 parameters provided prognostic information in univariate analysis (**table 4**).

**Table 3:** Baseline laboratory parameters.

	n	Min.	Lower Quartile	Median	Upper Quartile	95th P*.	Max.
**AFP (ng/ml)**	388	0.8	6.65	40.5	423.0	19788.0	577000.0
**Bilirubin (mg/dl)**	404	0.25	0.9	1.3	2.2	5.9	32.7
**Quick (%)**	402	35.0	65.0	75.0	85.0	100.0	125.0
**AP (U/l)**	341	31	95	142	209	421	1371
**Albumin (g/dl)**	378	1.4	3.3	3.8	4.2	4.8	5.1

(P* = percentile).

**Table 4:** Baseline laboratory parameters - Quartiles.

	n	Median survival [months]	95%-CI	p-value
**AFP**				overall <0.0001*
¼	97	29.7	19.6–38.8	¼ vs. ½: 0.777
½	97	28.4	21.3–38.2	½ vs. ¾: 0.001*
¾	97	14.4	10.0–17.2	¾ vs. 1: 0.017*
1	97	8.6	6.0–12.7	
**Bilirubin**				overall<0.0001*
¼	98	28.8	22.5–34.0	¼ vs. ½: 0.214
½	109	18.9	15.6–28.4	½ vs. ¾: 0.55
¾	98	17.2	13.9–22.9	¾ vs. 1: 0.004*
1	99	9.1	5.7–11.6	
**Quick**				overall 0.0215*
¼	117	14.0	9.8–23.1	¼ vs. ½: 0.195
½	91	14.1	9.8–16.9	½ vs. ¾: 0.021*
¾	98	25.3	15.2–32.7	¾ vs. 1: 0.371
1	96	23.4	16.8–30.8	
**Albumin**				overall <0.0001*
¼	88	9.2	6.2–14.1	¼ vs. ½: 0.37
½	93	13.5	10.5–18.3	½ vs. ¾: 0.133
¾	106	22.2	14.4–28.6	¾ vs. 1: 0.025*
1	91	31.4	21.1–38.2	
**AP**				overall <0.0001*
¼	85	32,7	26,8–38,8	¼ vs. ½:0,150
½	85	27,6	21,7–48,1	½ vs. ¾: 0,030*
¾	86	18,1	13,5–25,2	¾ vs. 1: <0,0001*
1	85	6,4	4,5–9,8	

(* = Statistically significant).

### Tumor related data

Tumor related data are summarized in **table 5**. 156 (38.5%) of all patients had a single tumor node, however only 4.7% of all patients had a single tumor smaller than 2 cm. On the other side, only 12.6% of all cases showed a tumor burden that involved more than 50% of the liver. One third of all patients (33.8%) had more than 3 tumor nodes. In contrast, tumor features related to a more advanced local involvement like distant metastasis, lymph-node involvement and macroscopic vascular invasion were present in the minority of cases (6.4%, 28.2% and 20.1%, respectively).

**Table 5:** Baseline tumor-associated parameters.

	n	%	Median survival [months]	95%-CI	p-value
**BCLC Tumor extension**					<0.0001*
[1] Singular ≤2 cm	19	4.7	47.4	23.1-	[1] vs. [2]: 0.376
[2] 3 ≤3 cm, singular ≤5 cm	84	20.8	48.8	23.1–79.8	[2] vs. [3]: p<0.0001*
[3] Multilocular/multifocal	191	47.3	18.6	15.6–24.1	[3] vs. [4]: p<0.0001*
[4] Portal vein infiltration, N1, M1	110	27.2	6.3	4.5–10.1	
**Number of tumor nodes**					<0.0001*
1	156	38.5	33.1	23.1–48.8	1 vs. 2: 0.108
2	74	18.3	24.1	21.1–32.7	2 vs. 3: 0.137
3	38	9.4	18.3	10.5–25.6	3 vs. 4: 0.024*
>3	137	33.8	9.8	7.1–13.7	
**Tumor burden (% of liver)**					<0.0001*
<50%	354	87.4	22.5	18.1–25.9	
>50%	51	12.6	3.6	2.3–7.0	
**Macroscopic vascular invasion**					<0.0001*
No	314	79.9	22.5	18.1–26.8	
Yes	79	20.1	6.0	4.4–10.5	
**Lymph nodes**					0.0436*
<1 cm	290	71.8	20.1	15.5–25.5	
>1 cm	114	28.2	15.8	11.8–20.3	
**Distant metastasis**					<0.0001*
No	378	93.6	20.1	16.1–23.1	
Yes	26	6.4	6.2	3.5–12.2	

(CI = confidence interval; * = statistically significant).

### Therapy

**Table 6** depicts the treatment modalities of the HCC patients, focusing on the primary mode of therapy. In total, only 24% of all patients received a potentially curative treatment option (resection, OLT and local ablation) as primary mode of therapy. The remaining 76% of patients received either palliative treatment modalities (n = 261) or were offered best supportive care (n = 47). TACE was by far the most frequent mode of primary therapy, more than half of the patients received this radiological intervention (215 patients; 53.1%). Local ablation was performed in 53 patients (13.1%). This treatment group included 14 patients receiving an unmated RFA, while 37 patients received a TACE session closely prior to the RFA, 2 patients were treated with PEI. In 47 cases (11.6%), no specific tumor therapy could be offered due to advanced tumor stage and/or liver insufficiency, respectively. 42 patients (10.4%) received a surgical resection following diagnosis of HCC, making this procedure the third most common initial mode of tumor directed therapy. Details concerning the distribution of patients according to the different staging systems in each treatment option and the change of treatment options over the past decade are shown in the **supporting information tables S9, S10**. Additionally, the prognosis of HCC patients according to the treatment modalities is shown in **figure S1**.

**Table 6:** Primary mode of therapy.

Therapy	n	%
TACE	215	53.1
Local ablation	53	13.1
BSC	47	11.6
Resection	42	10.4
Sorafenib	26	6.4
Tamoxifen	12	3.0
Chemotherapy	5	1.2
SIRT	3	0.7
OLT	2	0.5

(Local ablation = 37 TACE/RFA, 14 RFA, 2 PEI).

### Survival analysis and prognostic factors

**Figure 1:**
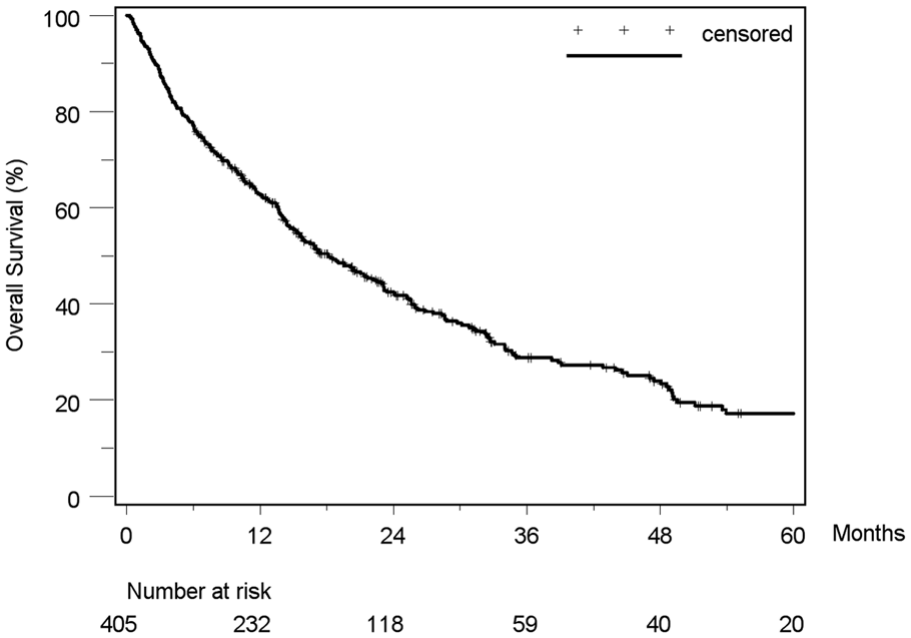
Kaplan-Meier estimated survival curve of 405 HCC-patients. Median survival was 18.1 months (95%-CI: 15.2–22.2). The 1-, 3-, and 5-year overall survival rates were 63%, 29% and 17%, respectively.

Median duration of follow-up was 14 months (range 0.2–113.1). By the end of follow-up in September 2009, 273/405 (67.4%) of the patients had died. Overall median survival was 18.1 months (95% CI: 15.2–22.2). The 1-, 3-, and 5-year overall survival rates were 63%, 29% and 17%, respectively (**figure 1**).

The following 16 parameters were associated with a significant impact on overall survival in *univariate* analysis: Clinical parameters (**table 2**): liver cirrhosis (p = 0.0417), ascites (p<0.0001), ECOG (p<0.0001), portal hypertension (p = 0.031), portal vein thrombosis (p<0.0001). Laboratory parameters (**table 4**): AFP (p<0.0001), bilirubin (p<0.0001), alkaline phosphatase (p<0.0001), Quick (p = 0.0215), albumin (p<0.0001). Tumor related parameters (**table 5**): BCLC-tumor extension (p<0.0001), number of tumor nodes (p<0.0001), tumor burden (p<0.0001), macroscopic vascular invasion (p<0.0001), lymph node involvement (p = 0.0436), distant metastasis (p<0.0001).

In *multivariate* analysis three laboratory parameters (AFP, bilirubin and aP), one clinical (ascites) and two tumor-related parameter (BCLC-tumor extension and number of tumor nodes), respectively remained significant predictors of survival (**table 7**).

**Table 7:** Significant prognostic parameters for overall survival in multivariate analysis.

	Hazard ratio for death	95% CI	P
**AFP**	1.098	1.062 to 1.135	<0.0001*
**Bilirubin**	1.612	1.345 to 1.932	<0.0001*
**Alkaline phosphatase**	1.494	1.256 to 1.777	<0.0001*
**Ascites**	1.534	1.258 to 1.870	<0.0001*
**Number of tumor nodes**	1.201	1.070 to 1.347	<0.0019*
**BCLC tumor features**	1.561	1.278 to 1.907	<0.0001*

(* = Statistically significant).

### Staging systems

**Figure 2:**
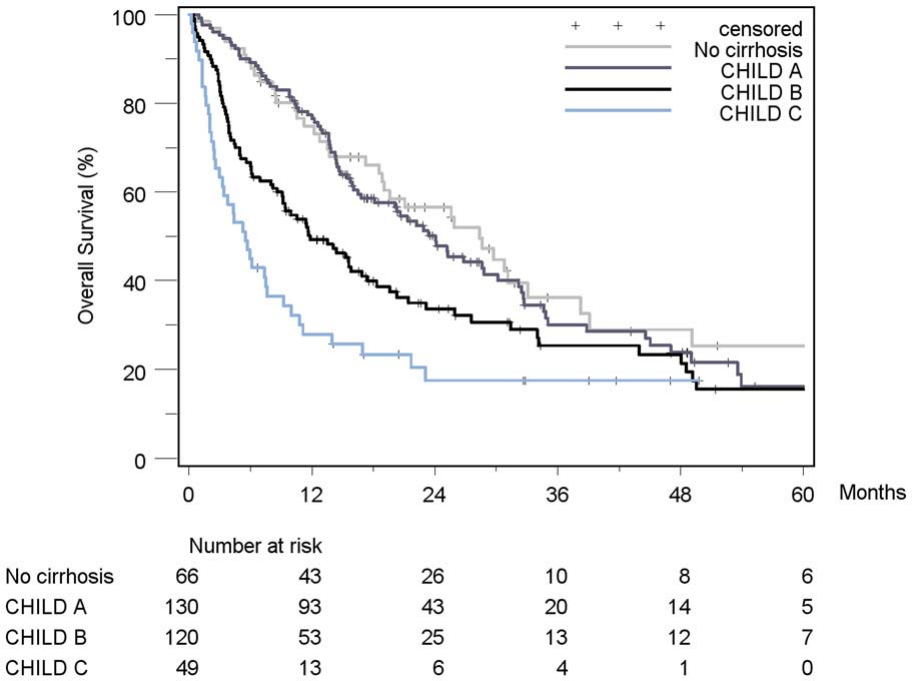
Kaplan-Meier survival analysis stratified according to the Child-Pugh-Score (n = 365). (No cirrhosis vs. Child A: p = 0.459; Child A vs. Child B: p = 0.009*; Child B vs. Child C: p = 0.016*).

Patient stratification and estimated median survival time according to the 7 staging systems are depicted in **table 8**. The majority of all patients were stratified to intermediate stages of the staging systems, the only exception being Okuda, which assigned over 50% of patients in the early stage I. None of the staging systems stratified the majority of patients into its respective advanced stage. When looking at the individual staging system as a whole, each showed a statistically significant association with prognosis. **Figures 2** **,** **3** **,** **4** **,** **5** **,** **6** **,** **7** **,** **8** show the Kaplan-Meier survival analysis stratified according to the 7 staging systems. The discriminatory ability of the staging systems was analyzed as well. All of the different strata in the Okuda, BCLC, GETCH, Child-Pugh and TNM-score characterized distinct survival groups (**figures 2** **,** **3** **,** **4** **,** **6** **and** **8**). The same was true for the CLIP-Score, except for its very early stage (CLIP 0 vs. CLIP 1: p = 0.262). 1- and 3-year survival with CLIP-score 1 was 80% and 40%, a CLIP-score of 2 had 1- and 3-year survival rates of 61% and 19% and a CLIP score of 3 was associated with a 1- and a 3-year survival of 40% und 13%, respectively. With a CLIP-score ≥4, 11% lived after 1 and only 5% after 3 years (**figure 5**). Analysis of the JIS-score revealed a lack of discriminatory ability between the early subcategories JIS 0 vs. JIS 1 (p = 0.233) and JIS 1 vs. JIS 2 (p = 0.391). Of note, patients without cirrhosis showed no difference in survival when compared to Child-A cirrhotic patients (p = 0.459).

**Figure 3:**
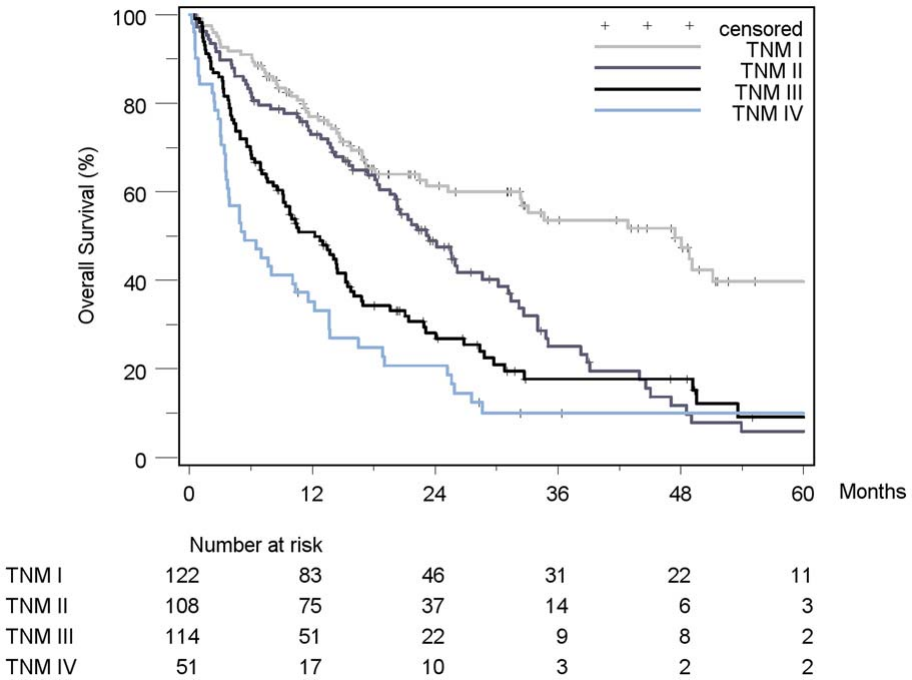
Kaplan-Meier survival analysis stratified according to the TNM-Staging System, 6^th^ edition (n = 395). (TNM I vs. TNM II: p<0.0001*; TNM II vs. TNM III: p = 0.012*; TNM III vs. TNM IV: p = 0.03*).

**Figure 4:**
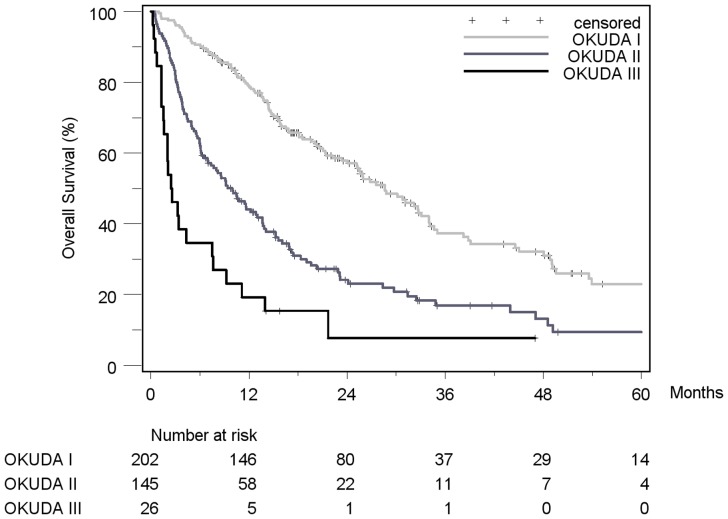
Kaplan-Meier survival analysis stratified according to the Okuda-Score (n = 373). (Okuda I vs. Okuda II: p<0.0001*; Okuda II vs. Okuda III: p = 0.001*).

**Figure 5:**
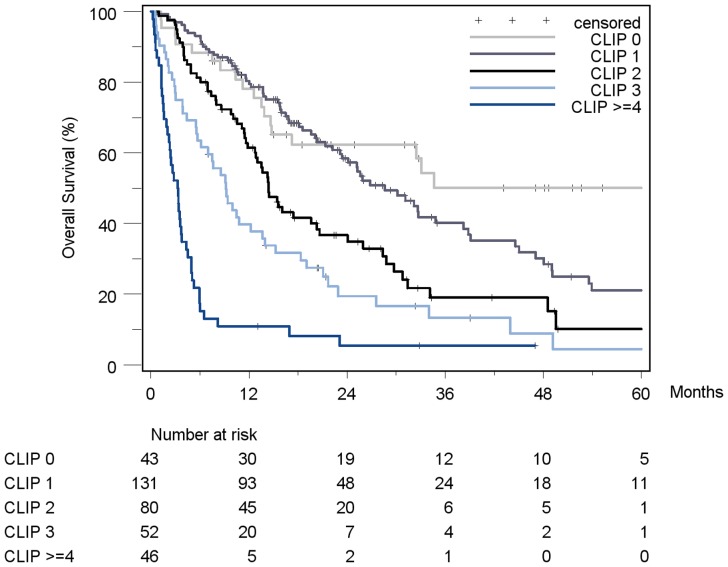
Kaplan-Meier survival analysis stratified according to the CLIP-Score (n = 352). (CLIP 0 vs. CLIP 1: p = 0.262; CLIP 1 vs. CLIP 2: p = 0.001*; CLIP 2 vs. CLIP 3: p = 0.023*; CLIP 3 vs. CLIP≥4: p = 0.005*).

**Figure 6:**
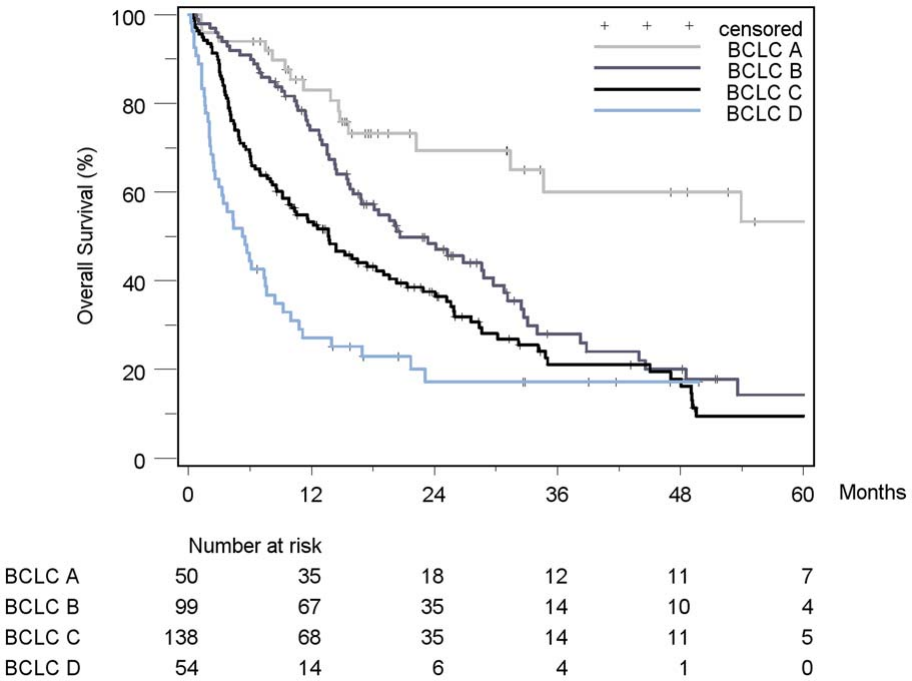
Kaplan-Meier survival analysis stratified according to the BCLC-Score (n = 341). (BCLC A vs. BCLC B: p = 0.001*; BCLC B vs. BCLC C: p = 0.018*; BCLC C vs. BCLC D: p = 0.005*).

**Figure 7:**
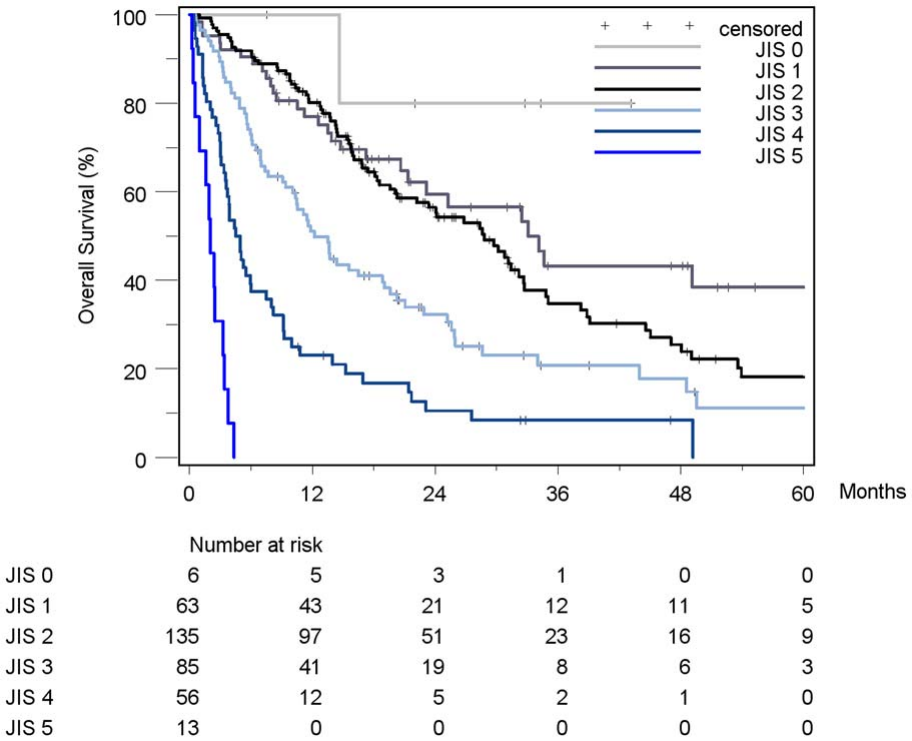
Kaplan-Meier survival analysis stratified according to the JIS-Score (n = 358). (JIS 0 vs. JIS 1: p = 0.233; JIS 1 vs. JIS 2: p = 0.391; JIS 2 vs. JIS 3: p<0.0001*; JIS 3 vs. JIS 4: p<0.0001*; JIS 4 vs. JIS 5: p<0.0001*).

**Figure 8:**
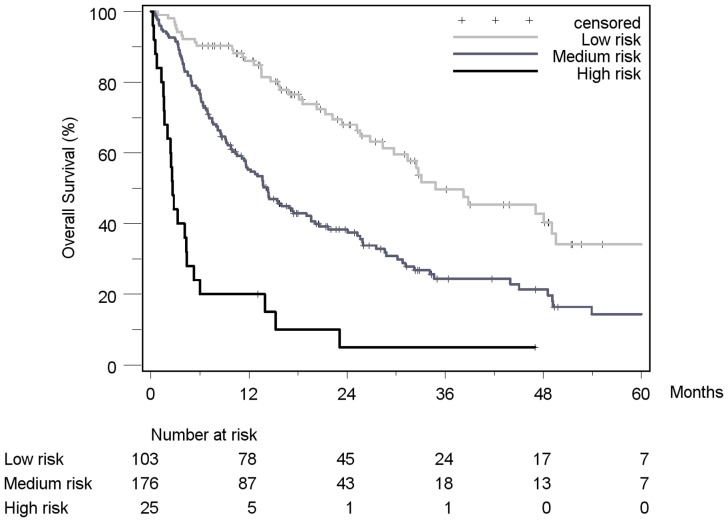
Kaplan-Meier survival analysis stratified according to the GETCH-Score (n = 304). (GETCH “low risk group” vs. “medium risk group”: p<0.0001*; GETCH “medium risk group” vs.“high risk group”: p<0.0001*).

**Table 8:** Patient distribution and estimated survival rates according to the seven staging systems.

Staging System	n	%	Median survival [months]	95%-CI	p-value
**Child-Pugh**					<0.0001*
No cirrhosis (nc)	66	18.1	28.4	18.9–38.2	nc vs. A: 0.469
A	130	35.6	24.1	16.8–30.1	A vs. B: 0.0009*
B	120	32.9	11.8	9.1–16.9	B vs. C: 0.016*
C	49	13.4	5.5	3.0–7.6	
**TNM (UICC 2010)**					<0.0001*
I	122	30.9	47.4	25.3–63.8	I vs. II: <0.0001*
II	108	27.3	23.4	19.6–30.1	II vs. III: 0.012*
III	114	28.9	12.2	9.1–15.2	III vs. IV: 0.03*
IV	51	12.9	5.4	3.5–11.6	
**Okuda**					<0.0001*
I	202	54.2	28.6	24.1–34.0	I vs. II: <0.0001*
II	145	38.9	10.0	6.9–13.0	II vs. III: 0.001*
III	26	7.0	2.5	1.6–7.5	
**CLIP**					<0.0001*
0	43	12.2	63.8	14.7–93.9	0 vs. 1: 0.262
1	131	37.2	28.6	23.1–38.2	1 vs. 2: 0.001*
2	80	22.7	14.4	11.8–20.3	2 vs. 3: 0.023*
3	52	14.8	9.2	5.7–13.7	3 vs. ≥4: 0.005*
≥4	46	13.1	3.3	2.0–3.8	
**BCLC**					<0.0001*
A	50	14.7	76.2	31.4-	A vs. B: 0.001*
B	99	29.0	20.6	15.8–29.7	B vs C: 0.018*
C	138	40.5	13.7	9.2–19.0	C vs. D: 0.005*
D	54	15.8	5.4	2.6–7.6	
**JIS**					<0.0001*
0	6	1.7		14.6-	0 vs. 1: 0.233
1	63	17.6	33.1	21.3–63.8	1 vs. 2: 0.391
2	135	37.7	28.8	20.3–32.7	2 vs. 3: <0.0001*
3	85	23.7	12.2	9.4–19.0	3 vs. 4: <0.0001*
4	56	15.6	4.7	3.5–6.0	4 vs. 5: <0.0001*
5	13	3.6	2.0	0.5–3.3	
**GETCH**					<0.0001*
Low	103	33.9	34.8	28.4–49.5	L vs. I: <0.0001*
Intermediate	176	57.9	14.2	11.6–19.0	I vs. H: <0.0001*
High	25	8.2	2.7	1.7–4.4	

(CI = Confidence interval; * = statistically significant).

### Comparison of the established staging systems

Further statistical analysis was performed in order to identify the staging system with the best predictive ability for survival. As shown in **tables 9** **and** **10**, ranking of the established staging systems based on the Akaike information criterion (AIC) and c-index resulted in identification of CLIP (AIC 2286, c-index 0.71) as the superior score for the examined HCC-cohort. Although confidence intervals of the c-index of CLIP and the other staging systems except for GETCH and Child-Pugh overlapped, there was a clear tendency towards a confirmation of the AIC results. JIS performed almost as well as CLIP, showing an AIC and c-index of 2293 and 0.70, respectively. The least suitable score was the uni-dimensional Child-Pugh-score (AIC 2369, c-index 0.63).

**Table 9:** Performance ranking of the staging systems based on the concordance-index (c-index).

Rank	Score	c-index	95% CI
**1**	CLIP	0.71	0.68 to 0.75
**2**	JIS	0.70	0.66 to 0.74
**3**	Okuda	0.66	0.63 to 0.69
**4**	BCLC	0.66	0.62 to 0.69
**5**	GETCH	0.64	0.61 to 0.67
**6**	TNM	0.64	0.60 to 0.68
**7**	Child	0.63	0.60 to 0.67

A higher c-index indicates better prognostic ability.

**Table 10:** Performance ranking of the staging systems based on the Akaike information criterion (AIC)..

Rank	Score	AIC-Score
**1**	CLIP	2286
**2**	JIS	2293
**3**	Okuda	2337
**4**	GETCH	2342
**5**	TNM	2342
**6**	BCLC	2343
**7**	Child	2369

A Lower AIC value indicates better prognostic ability.

## Discussion

### Characterization of study cohort

The performance of HCC staging systems always needs to be interpreted within the specific context of the examined study population. Therefore, an extensive characterization of the HCC-collective, going beyond the parameters needed for the staging systems, preceded the validation process in our study. The majority of patients were male (82.3%), and the median age of all patients was 63.4 years (range 27.8–84.8). These findings, as well as the fact that HCC predominantly arose in a cirrhotic liver (83.7%) are in line with most European HCC studies. In these studies, alcohol and HCV respectively have repeatedly been identified as the two leading etiologic factors for HCC in Europe [25], [26]. In our cohort of German HCC patients chronic alcohol abuse was the most frequent single risk factor (44%) followed by HCV (18.8%) supporting the data from a large study on epidemiology of HCC in southern Germany [27]. Over 40% of all HCC patients worldwide are Chinese [28]. Chinese HCC patients predominantly have an underlying HBV-infection and tend to be significantly younger than western patients due to transmission of the virus in younger years and its higher capability to promote tumor development in non-cirrhotic livers [29], [30]. Considering these major differences in epidemiology, it becomes clear why results of a staging system validation study in one geographic region cannot be automatically transferred to another. This comprehension is becoming increasingly acknowledged by investigators.

Many recent validation studies applied the staging systems to more selected groups of patients [15], [16], while our study included the whole range of tumor stages and their corresponding treatment options, from potentially curative treatment modalities (24%) to best supportive care (11.6%). The majority of patients were in a good or fairly good condition (92.6% ECOG 0–1) at time of diagnosis, which, despite the overall dismal prognosis, is a frequent finding in HCC [15]. TACE is considered the most widely-used palliative treatment option [31] and indeed was the primary mode of therapy in 53.1% of our patients, reflecting the common finding that most HCCs are detected in rather advanced stages [9]. In contrast to many other solid tumors, this is not so much related to distant metastasis (here only 6.4%) but more to *locally* advanced tumors as well as to the consequences of cirrhosis. The complex interplay of the tumor and the frequently underlying liver disease ultimately limits the range of applicable treatment options. In the literature about 30% of western HCC patients are reported to have potentially curable disease at time of diagnosis [32]. The slightly lower proportion in our cohort (24%) can be explained by the tertiary referral status of our center.

### Survival and prognostic factors

Overall median survival was 18.1 months and 5-year overall survival rate was 17%. Our survival data are comparable to another recent study from southern Germany, which showed an overall median survival of 19 months in a group that included more resectable HCC patients [27]. Reported survival rates for HCC vary significantly dependent on the examined study population. The broad range from 8 months in a largely non-surgical [26] and up to 64 months in a resectable group of patients [16] can in part be explained by the different degree of selection. Another reason for different survival data might be the bias of comparing different time periods. There is data suggesting that survival of HCC patients has improved over the past 3–4 decades, with five-year survival rates in the United States of approximately 4% in 1973 and 11.8% in 2001 [18]. This improvement might be attributed to better treatment options and surveillance programs, resulting in earlier detection of HCC [18].

Identification of prognostic factors within a given study population is the basis on which all staging systems have been developed. In the present study, a broad range of clinical, laboratory and tumor parameters showed statistical significance in *univariate* analysis. However, in *multivariate* analysis only aP, bilirubin, ascites, AFP, number of tumor nodes and BCLC-tumor extension remained strong predictors of survival. AFP, which is included only in 2 of the 7 examined staging systems (CLIP and GETCH), has repeatedly been identified as an independent prognostic factor in different settings [9], [33]–[34]. The current data emphasize the importance of AFP for prognostification in general and its exceptional role in screening, early detection and monitoring treatment is emphasized in a number of guidelines [35]. Except for TNM, bilirubin is included in all of the tested staging systems, underlining its outstanding prognostic relevance. In a large review of the literature, including a total of 23.968 patients from 72 studies bilirubin has been found to be under the six most important prognostic parameters [36]. Alkaline phosphatase (aP) is a less common prognostic marker of HCC. Of the currently tested staging systems, GETCH is the only one containing this parameter, nevertheless aP was identified as an independent prognostic factor, confirming the observations of Huitzil-Melendez et al. [15], which have been made in the context of an advanced HCC-collective. Ascites is included in the Child-Pugh, Okuda, BCLC, CLIP and JIS-scores. Therefore its significance in our multivariate analysis came as no surprise and is supported by many other studies showing its prognostic importance [37]. The tumor parameters included in the BCLC-score (“BCLC tumor features”) and the number of tumor nodes remained significant in multivariate analysis. Tumor parameters included in other staging systems, for example differentiating between tumor extension to more or less than 50% (part of the Okuda-score), are obviously not differentiated enough to bear an independent prognostic information. Altogether, the identification of three liver- as well as three tumor-related parameters as prognostic factors once again strengthens the need for a two-dimensional staging system including both categories. Some studies [16], [36] noted an independent prognostic meaning of the “general health status”. However, the consideration of this parameter in an ideal staging system as a “third dimension” as in BCLC (ECOG) and GETCH (Karnofsky) is not supported by our data.

### Validation and ranking of staging systems

A clear recommendation which staging system to choose for HCC patients, is of great importance for clinical decisions as well as planning of interventional studies [18]. There have been a number of studies to date focusing on the evaluation of staging systems [15], [16]. Although initially developed in different and inhomogeneous patient cohorts, some of the studies demonstrated a surprisingly good performance of the staging systems even in selected groups of HCC patients [16]. In our study, *all* of the tested staging systems and even the one-dimensional Child-Pugh and TNM showed a prognostic meaning (p<0.0001) when applied to the 405 HCC patients. On the one side, this is a sign of the excellent quality of the selected staging systems in general; on the other side this frequent observation underscores the basic problem with staging of HCC: With none of the scores totally failing and none standing out at first sight, more sophisticated measures are needed to identify the most suitable score. First of all, stratification of patients into the respective subcategories yielded further information in terms of discriminatory ability. All of the subcategories had distinct survival except for the early stages of CLIP (0 vs. 1) and JIS (0 vs. 1 and 1 vs. 2), an observation most likely a result of the underrepresentation of surgical patients in our cohort and not of a failure of these scores themselves, especially when considering the fact that CLIP (7 strata) and JIS (6 strata) represent the two most refined scores in terms of number of defined subgroups. In a study applying CLIP to surgical patients, the early stages in fact defined distinct survival groups [16]. An answer to the question which staging system should be preferred in a given HCC cohort cannot be obtained by simply comparing the performance of their respective strata. Established statistical methods to measure and compare the prognostic capability of a staging system are the AIC and c-index, respectively [22], [23]. AIC [38] and c-index [15] have been used in comparative HCC-staging system evaluation studies before, but to our knowledge, this is the first validation study to use both tools. The AIC as well as the c-index, provide information of the predictive accuracy of a staging system that exceed the information which can be derived by simply looking at the number of distinct strata of a staging system. The interpretation of c-index for instance is the probability that for a randomly chosen pair of patients the one with the higher prediction time is the one who survives longer. Thus the maximum achievable value for c is 1 regardless of the number of classes. The AIC is considered the most relevant reference for the comparison of different staging systems [38], which is why the current study considered it as the benchmark-test. When applied to our study cohort, both AIC and c-index consistently ranked CLIP as the superior score. However, the c-index of the CLIP score did show a non-overlapping confidence interval only with the inferior Child-Pugh and GETCH-sore. Nevertheless, there was a clear tendency to consistency with the AIC-results. This confirms the result of several validation studies from different geographic regions that ranked CLIP at number one [39]. Especially in patients undergoing nonsurgical therapy, CLIP seems to be the best staging system [15], [40]. CLIP was developed in a non-selected patient population, but had an emphasis on non-surgical patients [9], therefore it is known to have weaknesses in discriminating very early stages. Nevertheless, in some studies focusing on surgical patients it has also shown superior performance compared to other staging systems including BCLC [38]. Three out of six of the presently identified prognostic factors are included in the CLIP score (AFP, ascites and bilirubin), which might be an explanation for its superiority. On the other hand, BCLC also has three of the six parameters included (bilirubin, ascites and BCLC-tumor features) but demonstrated poorer values with regard to AIC and c-index. Although recommended by EASL and AASLD [13], [14] and obviously with good prognostic capability concerning the early stages [34], this is not the first time the BCLC staging system has performed in an inferior fashion in non-selected and especially in intermediate to advanced HCC patients [15]. The main advantage of BCLC over CLIP is its treatment algorithm, a tool that might simply be added to a revised CLIP as well to improve its practicability. With regard to AIC and c-index, JIS was consistently ranked at number 2 with only negligible differences when compared to CLIP. The good performance of this score, initially developed in Japan, is supported by previous studies [16], [41]; to our knowledge this is the first time it is being evaluated in a European HCC patient population. The least successful (with the highest AIC and lowest c-index) was the uni-dimensional Child-Pugh-score, which is lacking any tumor related parameter.

### Limitations

There are some potential limitations of this study. First, the retrospective fashion of the data collection resulted in a lack of data in some cases. Especially parameters like ECOG and HE are subject to interpretation and are more easily obtained in a prospective study. We tried to control this problem by applying standardized methods of obtaining these data. Furthermore, the good quality of our clinical database helped to retrieve all the necessary data, even retrospectively. Because of the clinical significance of the parameters needed for calculation of the scores, these values were available for most of the patients at time of diagnosis despite the retrospective character of this study. Second, relatively few patients were in the very early and early stages, limiting the value of our data for surgical cohorts and probably underestimating the prognostic capability of the TNM system, which is traditionally strong in surgical HCC patients. Finally, due to major differences in epidemiology as well as clinical and tumor parameters, applicability of our results obtained in a western HCC cohort to other geographic regions (i.e. Asia) is limited.

### Conclusion

In conclusion, our results indicate that in non-selected western HCC patients the Cancer of the Liver Italian Program-score (CLIP) (closely followed by JIS) is the best performing staging system among the seven currently used prognostic models.

## Supporting Information

Table S1 **Overview of Staging Systems.** Parameters included in the staging systems.
(DOCX)

Table S2 **Child-Pugh-Score.**
(DOCX)

Table S3 **TNM classification.**
(DOCX)

Table S4 **Okuda-Score.**
(DOCX)

Table S5 **CLIP-Score.**
(DOCX)

Table S6 **BCLC-Score.**
(DOCX)

Table S7 **JIS-Score.**
(DOCX)

Table S8 **GETCH-Score.**
(DOCX)

Table S9 **Patient distribution according to the different staging systems (and Child-Pugh) in each treatment option.** Shown are absolute numbers (and percentage) of the treatment modality within a specific stage. nc = no cirrhosis.
(DOCX)

Table S10 **Change of treatment modalities over time.** Absolute numbers (and percentage) with respect to the different time periods.
(DOCX)

Figure S1 **Prognosis of HCC patients according to the treatment modalities.** Overall survival was 43.9 months for local ablation (95% CI: 25.2–63.6), 34.0 months for surgical resection (95% CI: 17.2–93.8), 20.3 months for TACE (95% CI: 16–25.5), 9.1 months for Sorafenib (95% CI: 5.6–18.8) and 3.5 months for best supportive care (95% CI: 2.1–7.5).
(TIF)

## References

[1] Jemal A, Bray F, Center MM, Ferlay J, Ward E, et al (2011) Global cancer statistics. CA Cancer J Clin 6: 69–90. 10.3322/caac.20107 21296855

[2] Poon D, Anderson BO, Chen LT, Tanaka K, Lau WY, et al (2009) Management of hepatocellular carcinoma in Asia: consensus statement from the Asian Oncology Summit 2009. Lancet Oncol 10: 1111–8. 19880065 10.1016/S1470-2045(09)70241-4

[3] Sherman M (2005) Hepatocellular Carcinoma: Epidemiology, Risk Factors, and Screening. Semin Liver Dis 25: 143–154. 15918143 10.1055/s-2005-871194

[4] Ferlay J, Autier P, Boniol M, Heanue M, Colombet M, et al (2007) Estimates of the cancer incidence and mortality in Europe in 2006. Ann Oncol 18: 581–92. 17287242 10.1093/annonc/mdl498

[5] Lei HJ, Chau GY, Lui WY (2006) Prognostic value and clinical relevance of the 6th Edition 2002 American Joint Committee on Cancer staging system in patients with resectable hepatocellular carcinoma. J Am Coll Surg 203: 426–435. 17000385 10.1016/j.jamcollsurg.2006.06.030

[6] Pugh RN, Murray-Lyon IM, Dawson JL (1973) Transsection of the oesophagus for bleeding oesophageal varices. Br J Surg 60: 646–649. 4541913 10.1002/bjs.1800600817

[7] Okuda K, Ohtsuki T, Obata H (1985) Natural History of Hepatocellular Carcinoma and Prognosis in Relation to Treatment. Study of 850 Patients. Cancer 56: 918–928. 2990661 10.1002/1097-0142(19850815)56:4<918::aid-cncr2820560437>3.0.co;2-e

[8] Llovet JM, Brú C, Bruix J (1999) Prognosis of Hepatocellular Carcinoma: The BCLC Staging Classification. Semin Liver Dis 19: 329–338. 10518312 10.1055/s-2007-1007122

[9] The Cancer of the Liver Italian Program (CLIP) Investigators (1998) A New Prognostic System for Hepatocellular Carcinoma: A Retrospective Study of 435 Patients. Hepatology 28: 751–75. 9731568 10.1002/hep.510280322

[10] Chevret S, Trinchet JC, Mathieu D (1999) A new prognostic classification for predicting survival in patients with hepatocellular carcinoma. J Hepatol 31: 133–141. 10424293 10.1016/s0168-8278(99)80173-1

[11] Kudo M, Chung H, Osaki Y (2003) Prognostic staging system for hepatocellular carcinoma (CLIP score): its value and limitations, and a proposal for a new staging system, the Japan Integrated Staging Score (JIS score). J Gastroenterol 38: 207–215. 12673442 10.1007/s005350300038

[12] Forner A, Reig ME, de Lope CR, Bruix J (2010) Current strategy for staging and treatment: the BCLC update and future prospects. Semin Liver Dis 30: 61–74. 20175034 10.1055/s-0030-1247133

[13] Bruix J, Sherman M, Llovet JM, Beaugrand M, Lencioni R, et al (2001) Clinical management of hepatocellular carcinoma. Conclusions of the Barcelona-2000 EASL conference. European Association for the Study of the Liver. J Hepatol 35: 421–30. 11592607 10.1016/s0168-8278(01)00130-1

[14] Bruix J, Sherman M (2011) American Association for the Study of Liver Diseases. Management of hepatocellular carcinoma: an update. Hepatology 53: 1020–2. 21374666 10.1002/hep.24199 PMC3084991

[15] Huitzil-Melendez FD, Capanu M, O'Reilly EM, Duffy A, Gansukh B, et al (2010) Advanced hepatocellular carcinoma: which staging systems best predict prognosis? J Clin Oncol 28: 2889–95. 20458042 10.1200/JCO.2009.25.9895 PMC3651603

[16] Yang T, Zhang J, Lu JH, Yang LQ, Yang GS, et al (2011) A new staging system for resectable hepatocellular carcinoma: comparison with six existing staging systems in a large Chinese cohort. J Cancer Res Clin Oncol 137: 739–50. 20607551 10.1007/s00432-010-0935-3 PMC11827787

[17] Llovet JM, Ricci S, Mazzaferro V, Hilgard P, Gane, et al (2008) Sorafenib in advanced hepatocellular carcinoma. N Engl J Med 359: 378–390. 18650514 10.1056/NEJMoa0708857

[18] El-Serag HB (2011) Hepatocellular carcinoma. N Engl J Med 22: 1118–27. 10.1056/NEJMra1001683 21992124

[19] Bruix J, Sherman M (2005) Management of hepatocellular carcinoma. Practice Guidelines Committee, American Association for the Study of Liver Diseases. Hepatology 42: 1208–36. 16250051 10.1002/hep.20933

[20] Cash WJ, McConville P, McDermott E, McCormick PA, Callender ME, et al (2010) Current concepts in the assessment and treatment of hepatic encephalopathy. QJM 103: 9–16. 19903725 10.1093/qjmed/hcp152

[21] Buccheri G, Ferrigno D, Tamburini M (1996) Karnofsky and ECOG performance status scoring in lung cancer: a prospective, longitudinal study of 536 patients from a single institution. Eur J Cancer 32: 1135–41. 10.1016/0959-8049(95)00664-8 8758243

[22] Akaike H (1974) A new look at the statistical model identification. IEEE Trans Automat Contr 19: 716–723.

[23] Harrell FE Jr, Lee KL, Califf RM (1984) Regression modelling strategies for improved prognostic prediction. Stat Med 3: 143–152. 6463451 10.1002/sim.4780030207

[24] Kremers, WK. SAS macro. Available at http://mayoresearch.mayo.edu/mayo/research/biostat/upload/survcstd.sas. Accessed November 2, 2011.

[25] Greten TF, Papendorf F, Bleck JS (2005) Survival rate in patients with hepatocellular carcinoma: a retrospective analysis of 389 patients. Br J Cancer 92: 1862–1868. 15870713 10.1038/sj.bjc.6602590 PMC2361778

[26] Schöniger-Hekele, Müller C, Kutilek M (2001) Hepatocellular carcinoma in Central Europe: prognostic features and survival. Gut 48: 103–109. 11115830 10.1136/gut.48.1.103 PMC1728163

[27] Kirchner G, Kirovski G, Hebestreit A (2010) Epidemiology and survival of patients with hepatocellular carcinoma in Southern Germany. Int J Clin Exp Med 3: 169–179. 20607043 PMC2894652

[28] Skolnikk AA (1996) Armed with epidemiologic research, China launches programs to prevent liver cancer. JAMA 276: 1458. 8903245

[29] Yuen MF, Hou JL, Chutaputti A (2009) Hepatocellular carcinoma in the Asia pacific region. J Gastroenterol Hepatol 24: 346–353. 19220670 10.1111/j.1440-1746.2009.05784.x

[30] Li Q, Li H, Qin Y, Wang PP, Hao X (2007) Comparison of surgical outcomes for small hepatocellular carcinoma in patients with hepatitis B virus versus hepatitis C: A Chinese experience. J Gastroenterol Hepatol 22: 1936–1941. 17914973 10.1111/j.1440-1746.2006.04619.x

[31] Wang JH, Changchien CS, Hu TH (2008) The efficacy of treatment schedules according to Barcelona Clinic Liver Cancer staging for hepatocellular carcinoma – Survival analysis of 3892 patients. Eur J Cancer 44: 1000–1006. 18337087 10.1016/j.ejca.2008.02.018

[32] Llovet JM, Fuster J, Bruix J (2004) The Barcelona Approach: Diagnosis, Staging, and Treatment of Hepatocellular Carcinoma. Liver Transpl 10: 115–120. 14762851 10.1002/lt.20034

[33] Matsumo Y, Suzuki T, Asada I (1982) Clinical classification of hepatoma in Japan according to serial changes in serum alpha-fetoprotein levels. Cancer 49: 354. 6172192 10.1002/1097-0142(19820115)49:2<354::aid-cncr2820490224>3.0.co;2-j

[34] Grieco A, Pompili M, Caminiti G (2005) Prognostic factors for survival in patients with early-intermediate hepatocellular carcinoma undergoing non-surgical therapy: comparison of Okuda, CLIP, and BCLC staging systems in a single Italian centre. Gut 54: 411–418. 15710992 10.1136/gut.2004.048124 PMC1774422

[35] Lamerz R, Hayes P, Hoffmann RT, Löhe F, Taketa K (2010) National Academy of Clinical Biochemistry Laboratory Medicine Practice Guidelines for Use of Tumor markers in Liver Cancer. Clin Chem 56: e1–e48. 20207771 10.1373/clinchem.2009.133124

[36] Tandon P, Garcia-Tsao G (2009) Prognostic indicators in hepatocellular carcinoma: a systematic review of 72 studies. Liver Int 29: 502–510. 19141028 10.1111/j.1478-3231.2008.01957.x PMC2711257

[37] Tournoux-Facon C, Paoletti X, Barbare JC (2011) Development and validation of a new prognostic score of death for patients with hepatocellular carcinoma in palliative setting. J Hepatol 54: 108–114. 21047696 10.1016/j.jhep.2010.06.015

[38] Hsu CY, Hsia CY, Hang YH (2010) Selecting an Optimal Staging System for Hepatocellular Carcinoma: comparison of 5 currently used prognostic models. Cancer 116: 3006–3014. 20564406 10.1002/cncr.25044

[39] Levy I, Sherman M (2002) Liver Cancer Study Group of the University of Toronto (2002) Staging of hepatocellular carcinoma: assessment of the CLIP, Okuda, and Child-Pugh staging systems in a cohort of 257 patients in Toronto. Gut 50: 881–5. 12010894 10.1136/gut.50.6.881 PMC1773247

[40] Cho YK, Chung JW, Kim JK, Ahn YS, Kim MY, et al (2008) Comparison of 7 staging systems for patients with hepatocellular carcinoma undergoing transarterial chemoembolization. Cancer 112: 352–61. 18008352 10.1002/cncr.23185

[41] Toyoda H, Kumada T, Kiriyama S (2005) Comparison of the Usefulness of Three Staging Systems for Hepatocellular Carcinoma (CLIP, BCLC, and JIS) in Japan. Am J Gastroenterol 100: 1764–1771. 16086713 10.1111/j.1572-0241.2005.41943.x

