# Supplementary material for: Prognosis of Patients with Hepatocellular Carcinoma. Validation and Ranking of Established Staging-Systems in a Large Western HCC-Cohort
Source: PLoS One. 2012 Oct 5;7(10):e45066. doi: 10.1371/journal.pone.0045066 (PMC3465308; doi:10.1371/journal.pone.0045066)
Supplement: Table S1 — Overview of Staging Systems. Parameters included in the staging systems. (DOCX) [file pone.0045066.s001.docx]

| **Staging System** | **Tumor** | **Liver-function** | **General Health Status** |
| --- | --- | --- | --- |
| **Child-Pugh** | - | Bilirubin | - |
|  |  | Albumin |  |
|  |  | Quick |  |
|  |  | Ascites |  |
|  |  | Hepatic Encephalopathy |  |
| **TNM (UICC 2010)** | Number and size Tumor nodes | - | - |
|  | Vascular invasion |  |  |
|  | Lymph node metastasis |  |  |
|  | Distant metastasis |  |  |
| **Okuda** | </> 50% Tumorload | Bilirubin | - |
|  |  | Albumin |  |
|  |  | Ascites |  |
| **CLIP** | </> 50% Tumorload | Child-Pugh-Score | - |
|  | Singular or multiple nodes |  |  |
|  | AFP |  |  |
|  | Portal vein thrombosis |  |  |
| **BCLC** | Tumor extension | Child-Pugh-Score | Performance Status |
|  | Metastasis | Portal hypertension |  |
|  | Portal vein thrombosis | Bilirubin |  |
|  | Okuda-Score |  |  |
| **GETCH** | AFP | Bilirubin | Karnofsky-Index |
|  | Portal vein thrombosis | Alkaline phosphatase |  |
| **JIS** | TNM-Classification oft he LCSGJ (singular, < 2 cm, Vascular invasion, Lymph node metastasis) | Child-Pugh-Score | - |

Table S1: Overview of Staging Systems. Parameters included in the staging systems.
