# Supplementary material for: Prognosis of Patients with Hepatocellular Carcinoma. Validation and Ranking of Established Staging-Systems in a Large Western HCC-Cohort
Source: PLoS One. 2012 Oct 5;7(10):e45066. doi: 10.1371/journal.pone.0045066 (PMC3465308; doi:10.1371/journal.pone.0045066)
Supplement: Table S2 — Child-Pugh-Score. (DOCX) [file pone.0045066.s002.docx]

|  | 1 points | 2 points | 3 points |
| --- | --- | --- | --- |
| Bilirubin | < 2,0 mg/dl | 2,0-3,0 mg/dl | > 3,0 mg/dl |
| Albumin | > 3,5 g/dl | 2,8-3,5 g/dl | < 2,8 g/dl |
| Quick | > 70% | 70-40% | < 40% |
| Ascites (Ultrasound) | None | Moderate | Massive |
| Hepatic Encephalopathy | None | Grade I-II | Grade III-IV |
| Points | Score | | |
| 5-6 | A | | |
| 7-9 | B | | |
| 10-15 | C | | |

Table S2: Child-Pugh-Score.
