# Supplementary material for: Prognosis of Patients with Hepatocellular Carcinoma. Validation and Ranking of Established Staging-Systems in a Large Western HCC-Cohort
Source: PLoS One. 2012 Oct 5;7(10):e45066. doi: 10.1371/journal.pone.0045066 (PMC3465308; doi:10.1371/journal.pone.0045066)
Supplement: Table S3 — TNM classification. (DOCX) [file pone.0045066.s003.docx]

| **T1** | | Solitary tumor without vascular invasion |
| --- | --- | --- |
| **T2** | | Solitary tumor without vascular invasion, multiple tumors (none > 5 cm) |
| **T3** | | Multiple tumors > 5 cm or infiltration of a major branch of the portal vein or hepatic vein |
| **T4** | | Infiltration of adjacent structures (excluding gall bladder) |
|  |  | |
| **NX** | Cannot be assassed | |
| **N0** | No regional lymph node metastasis | |
| **N1** | Regional lymph node metastasis | |
|  | |  |
| **MX** | | Cannot be assassed |
| **M0** | | No distant metastasis |
| **M1** | | distant metastasis |
|  | |  |
| **Stage I** | | T1, N0, M0 |
| **Stage II** | | T2, N0, M0 |
| **Stage III** | | T3, N0, M0 or T4, N0, M0 |
| **Stage IV** | | Any T, N1, M0 (IVA) or any T, any N, M1 |

Table S3: TNM classification.
