# Supplementary material for: Prognosis of Patients with Hepatocellular Carcinoma. Validation and Ranking of Established Staging-Systems in a Large Western HCC-Cohort
Source: PLoS One. 2012 Oct 5;7(10):e45066. doi: 10.1371/journal.pone.0045066 (PMC3465308; doi:10.1371/journal.pone.0045066)
Supplement: Table S4 — Okuda-Score. (DOCX) [file pone.0045066.s004.docx]

|  | Positive | Negative |
| --- | --- | --- |
| Tumorextension | > 50 % | < 50% |
| Ascites | Clinically detectable | Clinically absent |
| Albumin | < 3 g/dl | > 3 g/dl |
| Bilirubin | > 3 mg/dl | < 3 mg/dl |
| Stage | | |
| I | No positive | |
| II | 1 or 2 positive | |
| III | 3 or 4 positive | |

Table S4: Okuda-Score.
