# Supplementary material for: Prognosis of Patients with Hepatocellular Carcinoma. Validation and Ranking of Established Staging-Systems in a Large Western HCC-Cohort
Source: PLoS One. 2012 Oct 5;7(10):e45066. doi: 10.1371/journal.pone.0045066 (PMC3465308; doi:10.1371/journal.pone.0045066)
Supplement: Table S5 — CLIP-Score. (DOCX) [file pone.0045066.s005.docx]

|  | **Score** |
| --- | --- |
| **Child-Pugh-Stage** |  |
| A | **0** |
| B | **1** |
| C | **2** |
| **Tumor-Morphology** |  |
| Uninodular and extension ≤ 50% | **0** |
| Multinodular and extension ≤ 50% | **1** |
| Massive or extension > 50% | **2** |
| **AFP** |  |
| < 400 ng/ml | **0** |
| ≥ 400 ng/ml | **1** |
| **Portal vein thrombosis** |  |
| No | **0** |
| Yes | **1** |

Table S5: CLIP-Score.
