# Supplementary material for: Prognosis of Patients with Hepatocellular Carcinoma. Validation and Ranking of Established Staging-Systems in a Large Western HCC-Cohort
Source: PLoS One. 2012 Oct 5;7(10):e45066. doi: 10.1371/journal.pone.0045066 (PMC3465308; doi:10.1371/journal.pone.0045066)
Supplement: Table S6 — BCLC-Score. (DOCX) [file pone.0045066.s006.docx]

| **Stage** | **Performance Status** | **Tumor Status** | | **Liver Function** |
| --- | --- | --- | --- | --- |
|  | | **Tumor Stage** | **Okuda Stage** |  |
| Stage A (early HCC) | | | | |
| **A1** | PST 0 | Single <5cm | Okuda I | No portal Hypertension, normal Bilirubin |
| **A2** | PST 0 | Single <5cm | Okuda I | Portal Hypertension, normal Bilirubin |
| **A3** | PST 0 | Single <5cm | Okuda I | Portal Hypertension, Abnormal Bilirubin |
| **A4** | PST 0 | 3 tumors < 3 cm | Okuda I-II | Child-Pugh A-B |
| Stage B (intermediate HCC) | | | | |
|  | PST 0 | Large, multinodular | Okuda I-II | Child-Pugh A-B |
| Stadium C (advanced HCC) | | | | |
|  | PST 1-2 | Vascular invasion or extrahepatic metastasis | Okuda I-II | Child-Pugh A-B |
| Stadium D (end-stage HCC) | | | | |
|  | PST 3-4 | Any | Okuda III | Child-Pugh C |
| **Treatment recommendations** | | | | |
| **Stage A** | Resection, Transplantation, RFA, PEI | | | |
| **Stage B** | TACE | | | |
| **Stage C** | Sorafenib | | | |
| **Stage D** | Supportive care | | | |

Table S6: BCLC-Score.
