# Supplementary material for: Prognosis of Patients with Hepatocellular Carcinoma. Validation and Ranking of Established Staging-Systems in a Large Western HCC-Cohort
Source: PLoS One. 2012 Oct 5;7(10):e45066. doi: 10.1371/journal.pone.0045066 (PMC3465308; doi:10.1371/journal.pone.0045066)
Supplement: Table S7 — JIS-Score. (DOCX) [file pone.0045066.s007.docx]

|  | **Scores** | | | |
| --- | --- | --- | --- | --- |
| **Score** | **0** | **1** | **2** | **3** |
| **Child-Pugh** | A | B | C | - |
| **TNM Classification (LCSGJ)** | I | II | III | IV |

Table S7: JIS-Score.
