# Supplementary material for: Prognosis of Patients with Hepatocellular Carcinoma. Validation and Ranking of Established Staging-Systems in a Large Western HCC-Cohort
Source: PLoS One. 2012 Oct 5;7(10):e45066. doi: 10.1371/journal.pone.0045066 (PMC3465308; doi:10.1371/journal.pone.0045066)
Supplement: Table S8 — GETCH-Score. (DOCX) [file pone.0045066.s008.docx]

| **Points** | **0** | **1** | **2** | **3** |
| --- | --- | --- | --- | --- |
| **Karnofsky-Index** | ≥ 80% |  |  | < 80% |
| **Bilirubin [μmol/l]** | < 50 |  |  | ≥ 50 |
| **AP (ULN)** | < 2 |  | ≥ 2 |  |
| **AFP [μg/l]** | < 35 |  | ≥ 35 |  |
| **Portal vein thrombosis** | No | Yes |  |  |
| Stage A (Low mortality risk): 0 points | | | | |
| Stage B (Intermediate mortality risk): 1-5 points | | | | |
| Stage C (Intermediate mortality risk): ≥ 6 points | | | | |

Table S8: GETCH-Score.
