# Supplementary material for: Prognosis of Patients with Hepatocellular Carcinoma. Validation and Ranking of Established Staging-Systems in a Large Western HCC-Cohort
Source: PLoS One. 2012 Oct 5;7(10):e45066. doi: 10.1371/journal.pone.0045066 (PMC3465308; doi:10.1371/journal.pone.0045066)
Supplement: Table S9 — Patient distribution according to the different staging systems (and Child-Pugh) in each treatment option. Shown are absolute numbers (and percentage) of the treatment modality within a specific stage. nc = no cirrhosis. (DOCX) [file pone.0045066.s009.docx]

| **Score** | **Stage** | **TACE** | **Local ablation** | **BSC** | **Resection** | **Sorafenib** | **Tamoxifen** | **Chemo** | **SIRT** | **OLT** |
| --- | --- | --- | --- | --- | --- | --- | --- | --- | --- | --- |
| CHILD | nc | 31 (47.0) | 2 ( 3.0) | 3 ( 4.5) | 18 (27.3) | 9 (13.6) | 2 ( 3.0) | 1 ( 1.5) |  |  |
| CHILD | A | 82 (63.1) | 21 (16.2) | 6 ( 4.6) | 8 ( 6.2) | 6 ( 4.6) | 3 ( 2.3) | 1 ( 0.8) | 3 ( 2.3) |  |
| CHILD | B | 59 (49.2) | 19 (15.8) | 19 (15.8) | 8 ( 6.7) | 7 ( 5.8) | 5 ( 4.2) | 2 ( 1.7) |  | 1 ( 0.8) |
| CHILD | C | 23 (46.9) | 2 ( 4.1) | 18 (36.7) |  | 2 ( 4.1) | 2 ( 4.1) | 1 ( 2.0) |  | 1 ( 2.0) |
|  | | | | | | | | | | |
| TNM | I | 48 (39.3) | 33 (27.0) | 5 ( 4.1) | 33 (27.0) | 2 ( 1.6) |  |  |  | 1 ( 0.8) |
| TNM | II | 77 (71.3) | 15 (13.9) | 6 ( 5.6) | 3 ( 2.8) | 4 ( 3.7) | 1 ( 0.9) | 1 ( 0.9) | 1 ( 0.9) |  |
| TNM | III | 63 (55.3) | 1 ( 0.9) | 20 (17.5) | 5 ( 4.4) | 12 (10.5) | 7 ( 6.1) | 3 ( 2.6) | 2 ( 1.8) | 1 ( 0.9) |
| TNM | IV | 20 (39.2) | 4 ( 7.8) | 15 (29.4) |  | 8 (15.7) | 3 ( 5.9) | 1 ( 2.0) |  |  |
|  | | | | | | | | | | |
| OKUDA | I | 119 (58.9) | 33 (16.3) | 9 ( 4.5) | 28 (13.9) | 9 ( 4.5) | 1 ( 0.5) |  | 3 ( 1.5) |  |
| OKUDA | II | 79 (54.5) | 12 ( 8.3) | 22 (15.2) | 5 ( 3.4) | 12 ( 8.3) | 9 ( 6.2) | 5 ( 3.4) |  | 1 ( 0.7) |
| OKUDA | III | 5 (19.2) | 1 ( 3.8) | 15 (57.7) | 1 ( 3.8) | 1 ( 3.8) | 2 ( 7.7) |  |  | 1 ( 3.8) |
|  | | | | | | | | | | |
| CLIP | 0 | 14 (32.6) | 10 (23.3) | 2 ( 4.7) | 14 (32.6) | 3 ( 7.0) |  |  |  |  |
| CLIP | 1 | 76 (58.0) | 25 (19.1) | 4 ( 3.1) | 15 (11.5) | 4 ( 3.1) | 2 ( 1.5) | 1 ( 0.8) | 3 ( 2.3) | 1 ( 0.8) |
| CLIP | 2 | 64 (80.0) | 2 ( 2.5) | 6 ( 7.5) | 1 ( 1.3) | 4 ( 5.0) | 3 ( 3.8) |  |  |  |
| CLIP | 3 | 23 (44.2) | 4 ( 7.7) | 11 (21.2) | 3 ( 5.8) | 7 (13.5) | 2 ( 3.8) | 2 ( 3.8) |  |  |
| CLIP | >=4 | 12 (26.1) | 1 ( 2.2) | 20 (43.5) |  | 5 (10.9) | 5 (10.9) | 2 ( 4.3) |  | 1 ( 2.2) |
|  | | | | | | | | | | |
| BCLC | A | 16 (32.0) | 18 (36.0) | 3 ( 6.0) | 12 (24.0) | 1 ( 2.0) |  |  |  |  |
| BCLC | B | 71 (71.7) | 8 ( 8.1) | 1 ( 1.0) | 13 (13.1) | 4 ( 4.0) |  | 1 ( 1.0) | 1 ( 1.0) |  |
| BCLC | C | 76 (55.1) | 15 (10.9) | 20 (14.5) | 2 ( 1.4) | 11 ( 8.0) | 8 ( 5.8) | 3 ( 2.2) | 2 ( 1.4) | 1 ( 0.7) |
| BCLC | D | 23 (42.6) | 2 ( 3.7) | 22 (40.7) | 1 ( 1.9) | 2 ( 3.7) | 2 ( 3.7) | 1 ( 1.9) |  | 1 ( 1.9) |
|  | | | | | | | | | | |
| JIS | 0 | 3 (50.0) | 3 (50.0) |  |  |  |  |  |  |  |
| JIS | 1 | 27 (42.9) | 12 (19.0) | 2 ( 3.2) | 20 (31.7) | 2 ( 3.2) |  |  |  |  |
| JIS | 2 | 87 (64.4) | 20 (14.8) | 5 ( 3.7) | 10 ( 7.4) | 6 ( 4.4) | 2 ( 1.5) | 1 ( 0.7) | 3 ( 2.2) | 1 ( 0.7) |
| JIS | 3 | 51 (60.0) | 6 ( 7.1) | 10 (11.8) | 3 ( 3.5) | 10 (11.8) | 4 ( 4.7) | 1 ( 1.2) |  |  |
| JIS | 4 | 21 (37.5) | 3 ( 5.4) | 19 (33.9) |  | 4 ( 7.1) | 5 ( 8.9) | 3 ( 5.4) |  | 1 ( 1.8) |
| JIS | 5 | 2 (15.4) |  | 9 (69.2) |  | 2 (15.4) |  |  |  |  |
|  | | | | | | | | | | |
| GETCH | Low | 56 (54.4) | 22 (21.4) | 3 ( 2.9) | 15 (14.6) | 3 ( 2.9) | 1 ( 1.0) | 1 ( 1.0) | 2 ( 1.9) |  |
| GETCH | Intermediate | 99 (56.3) | 18 (10.2) | 23 (13.1) | 11 ( 6.3) | 15 ( 8.5) | 5 ( 2.8) | 3 ( 1.7) | 1 ( 0.6) | 1 ( 0.6) |
| GETCH | High | 6 (24.0) | 1 ( 4.0) | 15 (60.0) |  | 1 ( 4.0) |  | 1 ( 4.0) |  | 1 ( 4.0) |

**Table S9: Patient distribution according to the different staging systems (and Child-Pugh) in each treatment option.** Shown are absolute numbers (and percentage) of the treatment modality within a specific stage. nc= no cirrhosis.
