# Supplementary material for: Prognosis of Patients with Hepatocellular Carcinoma. Validation and Ranking of Established Staging-Systems in a Large Western HCC-Cohort
Source: PLoS One. 2012 Oct 5;7(10):e45066. doi: 10.1371/journal.pone.0045066 (PMC3465308; doi:10.1371/journal.pone.0045066)
Supplement: Table S10 — Change of treatment modalities over time. Absolute numbers (and percentage) with respect to the different time periods. (DOCX) [file pone.0045066.s010.docx]

| **THERAPY** | **1998-2003** | **2003-2006** | ≥ **2007** |
| --- | --- | --- | --- |
| **TACE** | 42 (56.8) | 102 (61.4) | 71 (43.0) |
| **Local Ablation** | 6 ( 8.1) | 18 (10.8) | 29 (17.6) |
| **BSC** | 2 ( 2.7) | 22 (13.3) | 23 (13.9) |
| **Resection** | 13 (17.6) | 20 (12.0) | 9 ( 5.5) |
| **Sorafenib** |  |  | 26 (15.8) |
| **Tamoxifen** | 11 (14.9) | 1 ( 0.6) |  |
| **Chemotherapy** |  | 2 ( 1.2) | 3 ( 1.8) |
| **SIRT** |  |  | 3 ( 1.8) |
| **OLT** |  | 1 ( 0.6) | 1 ( 0.6) |

**Table S10: Change of treatment modalities over time.** Absolute numbers (and percentage) with respect to the different time periods.
